# New information of the biodiversity of the nymphalid family (Insecta, Lepidoptera, Nymphalidae) species collected in Romania’s fauna between 1887–1984

**DOI:** 10.3897/BDJ.11.e98737

**Published:** 2023-01-09

**Authors:** Cristina Stancă-Moise, George Moise, Tom Brereton, Mirela Stanciu

**Affiliations:** 1 "Lucian Blaga" University of Sibiu, Sibiu, Romania "Lucian Blaga" University of Sibiu Sibiu Romania; 2 Butterfly Conservation, Wareham, Dorset, United Kingdom Butterfly Conservation Wareham, Dorset United Kingdom

**Keywords:** butterfly, conservation, collections, endemic, Museum of Natural History in Sibiu

## Abstract

**Background:**

This paper complements the data published thus far about species of the nymphalid family with data collected in Romania’s eight regions between 1887 and 1984 and elsewhere in Europe (Austria, Germany, Croația, the Republic of Moldova, Serbia and Switzerland), including the date and the site of original collection. For the first time, this research presents the collecting information of the species held in the entomological collection of the Museum of Natural History in Sibiu. It identifies the species of the nymphalid family in six of the museum’s lepidoptera collections. These collections are of extraordinary interest not least because they are associated with natural scientists of European renown, such as Daniel Czekelius, Eugen Worell, Viktor Weindel, Rolf Weirauch, Heinrich Hann von Hannenheim and Eckbert Schneider. The analysis, cataloguing, centralisation and updating of the nomenclature resulted in a number of 1,865 specimens from 49 species and fifteen genera (of the 90 referenced in Romania’s fauna): *Aglais*, *Apatura*, *Araschnia*, *Argynnis*, *Brenthis*, *Boloria*, *Euphydryas*, *Inachis*, *Issoria*, *Libythea*, *Limenitis*, *Melitaea*, *Neptis*, *Nymphalis* and *Polygonia.* Data published in a previous article add 101 specimens from the *Vanessa* genus.

**New information:**

Most species originate regionally from the nine counties of Transylvania followed by Oltenia and Moldova (three counties each), Banat and Dobrogea (two counties each), Crișana, Satu Mare and Muntenia (one county each) and the capital of Romania, Bucharest. The species presented in this paper also include the extinct taxon *Polygoniaegea* (Cramer, 1775), Eugen Worrell collection and three species that are endemic to Romania: *Melitaearetyezatica* Diöszeghy, 1930, *Argynnispandoradacica* Hormuzaki, 1892, Daniel Czekelius collection and *Boloriapales* ([Denis & Schiffermüller], 1775) *carpathomeridionalis* Crosson et Popescu-Gorj, 1963, both in the Viktor Weindel collection.

## Introduction

Tracing the historical evolution of butterflies from the nymphalid family requires updating the information on the species’ long-term collection. Updating this information allows ascertaining in how far environmental and anthropogenic factors (Fox et al. 2019, Choi et al. 2021) have determined the temporal and spatial abundance and dominance of these species (Ștefanescu et al. 2021). In Romania, as in other European countries, the entomological material held at natural history museums is a valuable resource for researching declining or extinct populations of butterflies. In museums in Romania, data on butterfly species belonging to the nymphalid family have been collected over time from the whole country and the world and have been obtained through exchanges between specialists or from collectors. The data pertaining to specimens of the nymphalid family thus offer information about the collection sites (Popescu-Gorj 1960, Popescu-Gorj 1964, Popescu-Gorj and Drăhia 1964, Ciochia and Barbu 1980, Capușe and Kovács 1987, Burnaz 2002, Marcu and Rákosy 2002, Stănescu 2005, Chimișliu 2006, Chimișliu 2010, Chimișliu 2011, Chimișliu 2012). Since the 19^th^ century, fauna research in Transylvania has been indebted to the work of numerous professional and amateur entomologists with great love for nature. The following natural scientists founded the collections studied in this paper and analysed and determined the preserved material, thus bringing an important contribution to lepidopterological research: Daniel Czekelius (1856-1938, Sibiu); Arnold Müller (1884-1937, Sibiu); Eugen Worell (1884-1961, Sibiu), Viktor Weindel (1887-1966, Sibiu); Heinrich Hann von Hannenheim (1895-1971, Sibiu), Rolf Weirauch (1906-1984, Sibiu), Alexei Alexinschi (1899-1966, Iași); Hans Rebel (1861-1940, Vienna); László Diószeghy (1877-1942, Arad); Aurelian Popescu-Gorj (1914-1997, București) and Eckbert Schneider (1927) (Szèkely 2008). The Natural History Museum opened to the public in 1895. Owing to its priceless holdings, the Museum can be currently considered as an archive of lepidoptera biodiversity in Transylvania. It, therefore, plays a significant role in Romanian and European entomological research.

The Museum was founded by German-speaking Transylvanian-Saxon entomologists who collected material in the city of Sibiu, its surroundings, neighbouring regions and other Transylvanian locations (Czekelius 1897, Czekelius 1898, Worell 1951, Székely 1996, Székely 2003, Székely 2004), as well as in other geographical regions of Romania. Throughout time, several generations of natural scientists have evaluated and continue to evaluate the data held in these collections (Caradja 1931, Niculescu 1965, Schneider 1984, Chimișliu 1989, Schneider 1996, Rákosy et al. 2003, Moise 2011a, Moise 2011b, Moise 2011c, Rákosy 2013, Rakosy and Goia 2021). The Lepidoptera collections are of central importance to the Natural History Museum’s patrimony. To date, partial collecting data of the species have been published for the Daniel Czekelius, Eugen Worell, Viktor Weindel, Rolf Weirauch, Heinrich Hann von Hannenheim and Eckbert Schneider collections (Czekelius 1898, Rákosy et al. 2003, Török and Cuzepan 2014, Stancă-Moise 2017, Stancă-Moise 2018, Stancă-Moise 2019, Stancă-Moise 2020, Stancă-Moise 2021, Stancă-Moise 2022). The current paper evaluates unpublished data of specimens from the nymphalid family in order to assess this family’s historical distribution in Romanian and European fauna.

## Materials and methods

The material evaluated in this paper consists of specimens collected in Romania’s eight regions between 1887 and 1984. The analysis has also identified specimens collected in Austria, Germany, Croatia, Switzerland, the Republic of Moldova and Serbia. The species’ nomenclature was reviewed by using the actual identification keys as following (Rákosy et al. 2003, Szèkely 2008, Rákosy 2013, Rákosy et al. 2021) in conformity with the Fauna Europaea taxonomic system (Lampert 1923, Karsholt and Razowski 1996, Van Swaay and Warren 1999, Rakosy and Goia 2021). Each species is listed, indicating the examined material, collecting data (in the chronological order of years, months and days), number of specimens, collection sites (including the Romanian county and region), the conservation status (Rákosy et al. 2021) and name of the collector. Question marks are used in cases where information is missing, incomplete or the writing could not be deciphered. All data originate from museum labels; Romanian place names originally listed in German have been replaced with contemporary Romanian ones.

**Abbreviations**:

Collectors: Daniel Czekelius (DC), Eugen Worell (EW), Viktor Weindel (VW), Rolf Weirauch (RW), Heinrich Hann von Hannenheim (HH), Eckbert Schneider (ES).

Romanian regions: Banat (BT), Crișana (CR), Oltenia (OT), Muntenia (MT), Moldova (MD), Dobrogea (DB), Satu Mare (SM), Transilvania (TR)

Romania’s counties: Arad (AR), Alba (AB), Bistrița-Năsăud (BN), Brașov (BV), Caraș-Severin (CS), Cluj (CJ), Constanța (CT), Covasna (Cv), Galați (GL), Gorj (GJ), Harghita (HR), Hunedoara (HD), Ilfov (IF), Maramureș (MM), Mehedinți (MH), Mureș (MS), Neamț (NT), Satu Mare (SM), Sibiu (SB), Timiș (TM), Tulcea (TL), Vâlcea (VL), Vrancea (VN), București (B).

European countries: Austria (AU), Croația (HR), Germany (GE), Republic of Moldova (RMO), Serbia (RS), Switzerland (CH).

The conservation status: Extinct (EX), Critically endangered (CR), Endangered (EN), Vulnerable (VU), Near threatened (NT), Least concern (LC), Endemic (EN).

## Checklists

### Nymphalidae collected in Romania’s fauna between 1887 and 1984

#### Libythea celtis

(Laicharting, 1782)

712BE299-0967-567B-B3C7-268A247E89CA

##### Conservation status

EN

##### Distribution

RO

##### Notes

ES

#### Argynnis paphia

(Linnaeus, 1758)

BBC3CAF3-9FBC-5BF6-AC5E-25AA4214BB2F

##### Conservation status

NT

##### Distribution

RO, AU, RMO

##### Notes

DC, EW, VW, ES

#### Argynnis paphia f. valensina

[Esper, 1798]

F2784E02-C8CC-5642-A202-46D47F0CD613

##### Conservation status

NT

##### Distribution

RO, AU

##### Notes

EW

#### Argynnis pandora

([Denis & Schiffermüller], 1775)

6AEC3F5E-80FA-5698-B6A0-E02C8A0BAF61

##### Conservation status

VU

##### Distribution

RO

##### Notes

DC, EW, VW, RW, ES

#### Argynnis pandora dacica

Hormuzaki, 1892

750E049F-AD99-5EE1-87DD-95D6BA671D07

##### Conservation status

Endemic

##### Distribution

RO

##### Notes

EW

#### Argynnis (Speyeria) aglaja

(Linnaeus, 1758)

140BAC9A-9D62-5CFC-957F-F39A3649987A

##### Conservation status

LC

##### Distribution

RO

##### Notes

DC, EW, VW, RW

#### Argynnis adippe

(Denis & Schiffermüller, 1775)

BAA6E2A4-351F-50C9-B73D-425C551BD925

##### Conservation status

NT

##### Distribution

RO

##### Notes

DC, EW, VW

#### Argynnis (Fabriciana) niobef.cleodoxa

Esper 1789

2EE4629B-3152-5EE8-A0E3-A442460D6646

##### Conservation status

NT

##### Distribution

RO

##### Notes

VW

#### Argynnis (Fabriciana) niobe niobe

(Linnaeus, 1758)

7F193551-7681-59C8-BA7B-D03E88C9714A

##### Conservation status

NT

##### Distribution

RO, AU

##### Notes

DC, EW, VW, ES

#### Argynnis (Fabriciana) niobevar.pelopia

Borkhausen, 1788

5FDFE764-3602-5637-A32B-77220338BE50

##### Conservation status

NT

##### Distribution

RO

##### Notes

DC

#### Argynnis laodice

(Pallas, 1771)

0039CA02-9EBC-57BC-AB05-376067FBD11E

##### Conservation status

OUG 57/2007: 4B; IUCN: LC (Murariu and Maican 2021)

##### Distribution

RO

##### Notes

DC, EW, VW

#### Argynnis latonia var. hungarica

Aigner-Abafi, 1906

2D38C03E-89D4-5694-AE0E-79E0E1298A61

##### Conservation status

LC

##### Distribution

RO

##### Notes

DC

#### Issoria lathonia

(Linnaeus, 1758)

7B4BDEEB-64D4-5599-9163-2E016B8B593D

##### Conservation status

LC

##### Distribution

RO

##### Notes

DC, VW, HH, ES

#### Brenthis ino

Rottemburg, 1775

A4C7B511-3199-521A-8FCB-B3387DAE8490

##### Conservation status

VU

##### Distribution

RO

##### Notes

DC, VW

#### Brenthis daphne

([Denis & Schiffermüller], 1775)

18C952AB-FB4D-5CE4-8851-68F17921C48A

##### Conservation status

VU

##### Distribution

RO

##### Notes

DC, VW, ES

#### Brenthis hecate

([Denis & Schiffermüller], 1775)

3AEB1A85-E5E9-56DB-833D-9C63105853EC

##### Conservation status

VU

##### Distribution

RO

##### Notes

VW, HH, ES

#### Boloria euphrosyne

(Linnaeus, 1758)

F2708A60-936F-5346-A5F3-221F3F4705FC

##### Conservation status

VU

##### Distribution

RO

##### Notes

DC, VW, HH, ES

#### Boloria selene

([Denis & Schiffermüller], 1775)

535D70A7-6049-5AA0-B5B3-8FB55A29ED2D

##### Conservation status

NT

##### Distribution

RO

##### Notes

DC, VW, HH, ES

#### Boloria dia

(Linnaeus, 1767)

A87C6F0A-5484-5553-9E5F-F287DF1EAB58

##### Conservation status

LC

##### Distribution

RO

##### Notes

DC, VW, HH, ES

#### Boloria pales carpathomeridionalis

([Denis & Schiffermüller], 1775) Crosson & Popescu-Gorj, 1963

596F95A2-BAA9-5171-819C-73BEBC7C8814

##### Conservation status

Endemic

##### Distribution

RO

##### Notes

VW, HH

#### Inachis (Aglais) io

(Linnaeus, 1758)

2F7E7ADA-889C-589A-86FD-7A97FC105FAD

##### Conservation status

LC

##### Distribution

RO, RMO

##### Notes

DC, EW, VW, HH, RW, ES

#### Aglais urticae

(Linnaeus, 1758)

57962061-12A0-555D-B630-BD063BD20278

##### Conservation status

NT

##### Distribution

RO, RMO

##### Notes

DC, EW, VW, RW, ES

#### Polygonia c-album

(Linnaeus, 1758)

A06CF910-C236-509F-BB36-010F3731A390

##### Conservation status

NT

##### Distribution

RO, RMO

##### Notes

DC, EW, VW, HH, RW, ES

#### Polygonia egea

(Cramer, 1775)

9A2E99B8-F0D8-5A25-B65E-36405B617EF1

##### Conservation status

EX

##### Distribution

HR

##### Notes

EW

#### Araschnia levana

(Linnaeus, 1758)

D3E722DC-9506-5D45-B832-B3BA4A070899

##### Conservation status

NT

##### Distribution

RO, GE

##### Notes

DC, EW, VW, HH, RW, ES

#### Araschnia levana f. porima

Ochsenheimer, 1807

EE8A9D60-EC7D-51FB-B969-B201C6CFBC66

##### Conservation status

NT

##### Distribution

RO

##### Notes

DC

#### Araschnia levana f. prorsa

Linnaeus, 1758

003B52E7-3ADC-5DF0-8750-30F9BFDFDF91

##### Conservation status

NT

##### Distribution

RO

##### Notes

DC, EW, VW

#### Araschnia levana f. intermedia

Stichel, 1906

4D3D3472-7D09-5FFA-8018-720A521C4D89

##### Conservation status

NT

##### Distribution

RO

##### Notes

DC

#### Nymphalis antiopa

(Linnaeus, 1758)

B685CC7D-CBBB-5B0D-ADCC-A3B2FF4752E9

##### Conservation status

VU

##### Distribution

RO

##### Notes

DC, EW, VW, HH, RW, ES

#### Nymphalis polychloros

(Linnaeus, 1758)

96DD0376-9A76-5D22-AB1F-F1AD1AB6F49E

##### Conservation status

EN

##### Distribution

RO

##### Notes

DC, EW, VW, RW, ES

#### Nymphalis xanthomelas

([Denis & (Schiffermüller], 1775)

70ED3F29-66B6-5DFA-8D0F-D8E2A68C1712

##### Conservation status

IUCN: LC (Murariu and Maican 2021)

##### Distribution

Ro

##### Notes

DC, EW, VW, RW

#### Nymphalis vaualbum

([Denis & Schiffermüller], 1775)

75410177-7E91-524C-81F4-B5E640E72577

##### Conservation status

ONG 57/2007: 3, 4A, DH: II, IV; IUCN: LC (Murariu and Maican 2021)

##### Distribution

RO

##### Notes

DC, VW, RW

#### Euphydryas maturna

(Linnaeus, 1758)

0DE8A529-1E85-5D37-9EA1-0879B8253924

##### Conservation status

ONG 57/2007: 3, 4A, DH: II, IV, IUCN: DD (Murariu and Maican 2021)

##### Distribution

RO

##### Notes

DC, EW, ES

#### Euphydryas orientalis

(Herrich-Schaffer, [1851])

E5C9A171-0FEF-5EFE-BA4B-19F4B01C6ED6

##### Conservation status

CR

##### Distribution

RO

##### Notes

RW

#### Euphydryas aurinia

(Rottemburg, 1775)

C3FAFF5A-4A6B-5F92-8187-D211CB3092EF

##### Conservation status

ONG 57/2007: 3, 4A, DH: II (Murariu and Maican 2021)

##### Distribution

RO

##### Notes

DC, EW, VW, ES

#### Melitaea cinxia

(Linnaeus, 1758)

A4794F46-BB83-5B37-8BC5-98E71579102A

##### Conservation status

NT

##### Distribution

RO

##### Notes

DC, EW, VW, HH, RW, ES

#### Melitaea phoebe

([Denis & Schiffermüller], 1775)

4047EC2F-01CE-5D1B-AB34-3240D7CCE67B

##### Conservation status

NT

##### Distribution

RO, RMO

##### Notes

DC, EW, VW, RW, ES

#### Melitaea trivia

([Denis & Schiffermüller], 1775)

E9BF1AAA-3D0B-53EF-BEED-82B69164A82E

##### Conservation status

NT

##### Distribution

RO, RMO

##### Notes

DC, EW, VW, ES

#### Melitaea didyma

(Esper, 1778)

4FF99F90-5309-564A-A5FC-C5B148FB9FB9

##### Conservation status

LC

##### Distribution

RO, AU

##### Notes

DC, EW, VW, HH, RW

#### Melitaea didyma alpina

Staudinger, 1861

C3117AD9-53C3-5082-A1E2-A837AEB17E0B

##### Conservation status

VU

##### Distribution

RO

##### Notes

EW

#### Melitaea didyma occidentalis

Staudinger, 1861

0F1979CF-8839-5093-B959-F24FA988A935

##### Conservation status

VU

##### Distribution

RO

##### Notes

RW

#### Melitaea didyma meridionalis

Staudinger, 1870

F37F8322-EE97-5FE3-A14F-210276674962

##### Conservation status

VU

##### Distribution

RO

##### Notes

EW

#### Melitaea didymoides

Eversmann, 1847

19335340-B230-5B9B-90F9-911411A5C59C

##### Conservation status

VU

##### Distribution

RMO

##### Notes

EW

#### Melitaea dictynna

Esper, 1778

78990EA3-1A0B-510B-A4B9-BD003382B96A

##### Conservation status

VU

##### Distribution

RO

##### Notes

DC, EW, RW

#### Melitaea aurelia

Nickerl, 1850

0BA32BA6-F8A1-5CAD-BBCE-6AA1A2D73570

##### Conservation status

VU

##### Distribution

RO, RS

##### Notes

DC, EW, VW, HH, RW, ES

#### Melitaea athalia

(Rottemburg, 1775)

5A1FB6B2-732A-53DA-BAC7-F56B5ECC71DD

##### Conservation status

NT

##### Distribution

RO, RMO

##### Notes

DC, EW, VW, HH, RW, ES

#### Melitaea athalia mehadiensis

Gerhard, 1822

0D35EA98-BB3C-5AF0-BE6B-FAF59349C5FD

##### Conservation status

NT

##### Distribution

RO

##### Notes

EW, RW

#### Melitaea britomartis

Assmann, 1847

E9E5050A-5B58-5B9E-8143-0C43A2183E13

##### Conservation status

NT

##### Distribution

RO

##### Notes

RW

#### Melitaea diamina alpestris

Fruhstorfer, 1917

43BC2528-DE7E-5814-9165-6E210E25A145

##### Conservation status

NT

##### Distribution

RO

##### Notes

DC

#### Melitaea nana

Rehfons, 1910

18E62C71-ACCC-59FA-97EC-0556150D8A3B

##### Conservation status

VU

##### Distribution

RO

##### Notes

DC

#### Melitaea parthenoides

Keferstein, 1851

575D35B8-580E-5917-A4B2-4BF62B0AF05D

##### Conservation status

VU

##### Distribution

RO

##### Notes

DC, EW

#### Melitaea parthenie var. varia

Meyer-Dűr, 1851

A10AEC62-5BF1-5CB6-A036-E4654D5CD9BD

##### Conservation status

VU

##### Distribution

AU

##### Notes

EW

#### Melitaea retyezatica

Diöszeghy, 1930

5D1B5CD2-247A-5AA6-A287-72994ED9E355

##### Conservation status

Endemic

##### Distribution

RO

##### Notes

DC

#### Melitaea asteria

Freyer, 1828

C5CF5101-3EAB-53B8-9DEE-3D03DB48E4DE

##### Conservation status

VU

##### Distribution

RO

##### Notes

EW

#### Melitaea athalia dictynnoides

Hormuzaki, 1898

BB8C48BD-E921-5D9A-97B4-CB189EDC00BA

##### Conservation status

NT

##### Distribution

RO

##### Notes

DC

#### Limenitis populi

(Linnaeus, 1758)

968DE45F-B615-53D7-B484-BE4EF0883528

##### Conservation status

VU

##### Distribution

RO

##### Notes

DC, EW, VW, HH, RW

#### Limenitis populi var. tremulae

Esper, 1800

DD926DC0-8BBE-5EB5-AA46-6C35208A34DA

##### Conservation status

VU

##### Distribution

RO

##### Notes

DC, EW, VW, RW

#### Limenitis camilla

(Linnaeus, 1764)

0B1A9DFD-51A4-5DB0-96F1-9A827068D13B

##### Conservation status

VU

##### Distribution

RO

##### Notes

VW, RW

#### Limenitis sibilla (camilla)

Linnaeus, 176

AB282179-EBC3-5D95-BD0A-C3CA032640CE

##### Conservation status

VU

##### Distribution

RO, CH

##### Notes

DC, EW,

#### Limenitis reducta reducta

Staudinger, 1901

C100F37A-1141-5D78-A105-BB187627CA0F

##### Conservation status

EN

##### Distribution

RO

##### Notes

RW

#### Neptis aceris

sensu Lhomme, 1924

D6047C03-84A9-58DF-A18C-171FBE66DF71

##### Conservation status

VU

##### Distribution

RO

##### Notes

DC, HH

#### Neptis rivularis

(Scopoli, 1763)

8346C0BC-3B06-5392-8A63-7A35F7EF2691

##### Conservation status

NT

##### Distribution

RO

##### Notes

EW, VW, RW, ES

#### Neptis rivularis ludmila

Nordmann, 1851

5A4F5E37-A04C-58B7-AF74-76D5A2E791A3

##### Conservation status

NT

##### Distribution

RO

##### Notes

VW

#### Neptis sappho

Pallas, 1771

9D61110B-586D-56B9-BACA-543C8A1DCC76

##### Conservation status

VU

##### Distribution

RO

##### Notes

VW, RW, ES

#### Neptis lucilla

([Denis & Schiffermüller], 1775)

5283B693-74CF-5592-B7F2-64ADBF27C5CB

##### Conservation status

NT

##### Distribution

RO

##### Notes

DC, EW, HH, RW, ES

#### Apatura ilia

([Denis & Schiffermüller], 1775)

3A654A24-7B38-5944-9AA7-6A80D0EC4C62

##### Conservation status

VU

##### Distribution

RO

##### Notes

DC, EW, VW, RW

#### Apatura clytie

([Denis & Schiffermüller], 1775)

0C9D4489-4625-5214-882A-CF730059A304

##### Conservation status

VU

##### Distribution

RO

##### Notes

DC, VW, RW

#### Apatura ilia var. eos

Rossi, 1794

2226EBCE-1F65-5F00-94EA-A62C68D784AD

##### Conservation status

VU

##### Distribution

RO

##### Notes

DC, EW, HH, ES

#### Apatura iris

(Linnaeus, 1758)

9C7C8FE0-6D26-59DF-AFE5-ACA00C2969D8

##### Conservation status

VU

##### Distribution

RO

##### Notes

DC, EW, VW, HH, ES

#### Apatura metis

Freyer, 1829

EA5D0E3F-A5DF-5910-B6D6-99E4F07533CC

##### Conservation status

VU

##### Distribution

RO

##### Notes

EW

## Discussion

**Figure 1.**
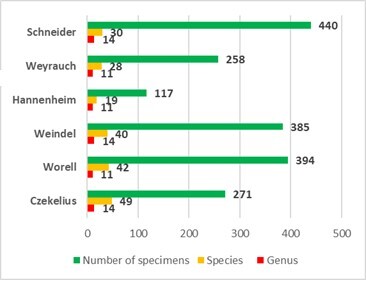
Systematic representation of the six collections by genera, species and number of specimens.

The analysis of the six collections of lepidoptera under scrutiny in this article focused on 1,865 specimens collected between 1887 and 1984 and identified 49 species and subspecies of the 90 mentioned in Romania’s fauna (Rákosy 2013, Rakosy and Goia 2021). Earlier data regarding the *Vanessa* genus are based on 101 specimens, 52 specimens of *V.atalanta* and 49 specimens of *V.cardui* (Stancă-Moise 2022). The species’ listing for each collection (Fig. 1) includes the following results: Daniel Czekelius’s collection, with 271 specimens, 14 genera and 49 (Table 1), Eugen Worrell’s collection, 11 genera and 41 species (Table 2), Viktor Weindel’s collection, 14 genera and 40 species (Table 3), Heinrich Hann von Hannenheim’s collection, 11 genera, 19 species (Table 4), Rolf Weyrauch’s collection, 11 genera and 28 species (Table 5) and Eckbert Schneider's collection with 14 genera and 30 species (Table 6). The oldest specimens are *Boloriaselene* ([Denis & Schiffermüller], 1775) and *Nymphalispolychloros* (Linnaeus, 1758) and were collected 135 years ago. The collectors specified on the labels are Daniel Czekelius, Béla Kiss, Arnold Müller, László Diószeghy, Friedrich Deubel, Paul Tiltscher, Miklos Tűtscher, Kimakowics, Adalbert Prall, Karl Alberti, Karl Petri and Vincenz Kollar.

Table 1

Daniel Czekelius’s collection of lepidoptera is the oldest with specimens collected between 1887 and 1934 in the regions SM and TR, Sibiu County and nearby locations. This collection consists of 14 genera and 49 species represented by a number of 271 specimens (Fig. 1) and is, thus, the most complex collection regarding the species’ biodiversity (Table 1). This collection also includes an endemic species, *Melitaearetyezatica* Diöszeghy, 1930 from *Melitaeaathalia* (Rottemburg, 1775), represented by twenty-six specimens collected by László Diószeghy in the Retezat Mountains (Southern Carpathians) between 1919 and 1922. The species in this collection represent the following conservation status: 18 taxa VU- 36.,74%, 18 taxa NT- 36.74%, six tax LC- 12.24%, four taxa EN-8.16%, two taxa CR-4.08% and one endemic taxon, 2.04%.

Eugen Worell’s collection consists of 394 specimens of 11 genera and 42 species collected between 1888 and 1959 (Fig. 1). The species originate from Romania’s five regions (BT, DB, OT, MD, MT and TR) and other European countries (Austria, Croatia, Germany, the Republic of Moldova and Switzerland). The oldest specimen is the *Nymphalispolychloros* (Linnaeus, 1758) species and was collected in 1888. The collectors listed on the labels are Eugen Worell, Daniel Czekelius, László Diószeghy and Alexei Alexinschi. Considering their conservation status, these species are grouped as following: one species EX-2.38%, Polygoniaegea (Cramer, 1775), from the site Pula, Istria, Croația, one species CR-2.38%, three species EN-7.15%, 19 species VU-45.23%, 15 species NT-35.71% and three species LC-7.15% (Table 2).

Viktor Weindel’s collection contains 385 specimens of 14 genera and 40 species of butterflies of the nymphalid family (Fig. 1). They were collected in OT and TR regions between 1903 and 1958, from various sites in Sibiu County and the Făgăraș Mountains. The oldest specimen *Argynnispaphia* (Linnaeus, 1758) was collected in 1903. The collection also contains an endemic species, *Boloriapales* ([Denis & Schiffermüller], 1775) *carpathomeridionalis* Crosson et Popescu-Gorj, 1963 that was collected in the Făgăraș Mountains. Viktor Weindel is the collector listed on the labels. The endangerment level of the specimens is the following: two species CR-5%, three species EN-7.5%, 15 species VU-37.5%, 15 species NT-37.5% and five species LC-12.5% (Table 3).

The fewest specimens are found in Heinrich Hann von Hannenheim’s collection consisting of 117 specimens distributed in 11 genera and 19 species (Fig. 1). The oldest specimen is *Brentishecates* ([Denis & Schiffermüller], 1775) collected in 1928. The species were collected in BT, DB and TR and were determined by the collector himself whose name can be found on all labels. Conservation status is as follows: nine species VU-47.37%, six species NT-31.58% and four species LC-21.05% (Table 4).

Rolf Weihrauch’s collection contains 258 specimens distributed in 11 genera and 28 species (Figure 1) collected from 1922 to 1978. The oldest specimen is *Euphydryasorientalis* (Herrich-Schaffer, [1851]) sin. *Euphydryasaurinia* (Rottemburg, 1775) collected in 1922. The information on the labels is unreadable. The species were collected in BT, DB, OT, MD, MT and TR from the Southern Carpathians, as well as in Austria. All species were determined by the collector whose name appears on all labels. The analysis of their endangerment level is as follows: three taxa CR-10.7%, two taxa EN-7.14%, 11 taxa VU-39.28%, 10 taxa NT-35.73% and two taxa LC-7.14% (Table 5).

The newest is Eckbert Schneider’s collection. It contains 440 specimens of 14 genera and 30 species (Fig. 1) collected between 1949 and 1984 in BT, CR, DB, OT, MD and TR, as well as the Western Carpathians. The oldest specimen was collected in 1949 and is the only *Lybiteacelsis* (Laicharting, 1782) species held by the Museum. The endangerment level of the collection’s specimens is as follows: three specimens EN-10%, 10 specimens VU-33.33%, 12 specimens NT-40% and five specimens LC-16.67% (Table 6).

We present data pertaining to genus diversity, species and existing specimen number present in each collection (Fig. 1). The oldest collection (DC) displays the highest diversity of species. This collection contains 14 genera and 49 species. It is followed in chronological order by EW with 42 species and VW with 40, confirming that, at that time, natural scientists were interested in learning more about and studying the biodiversity of butterflies in SB, the neighbouring localities, as well as in other TR sites and Romanian regions. The species from the more recent collections (HH and RW) contain a lesser number of genera and species, but include more specimens from the DB region. The newest collection (ES) re-confirms the presence of 30 butterfly species of the Nymphalid family captured in six Romanian regions (TR, BT, CR, MD, DB and OT) that are also represented by the largest number of collected specimens.

**Figure 2.**
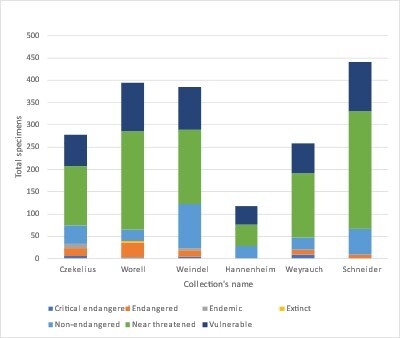
The distribution of species from the collections by IUCN categories.

To facilitate national and international comparison, we present data referring to the distribution of species onsidering the IUCN conservation categories in Fig. 2 (Rákosy 2013, Rakosy and Goia 2021), revealing that the majority of the species of all six collections are potentially endangered taxa (NT) followed by non-endangered (LC), vulnerable (VU), critically endangered (EN) extinct (EX) and endemic taxa.

**Figure 3.**
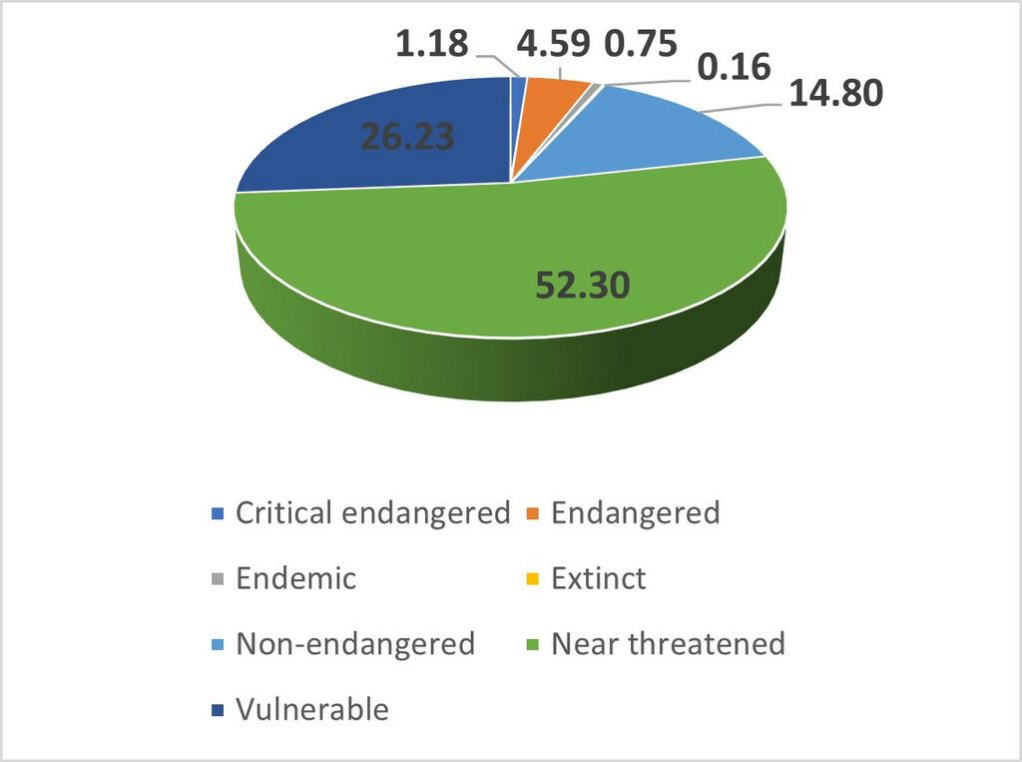
Nymphalid species and their evaluation based on Romania’s Red List (Rákosy et al. 2021, Rakosy and Goia 2021).

We also present the percentages of the Nymphalid family’s sozological categories in (Fig. 3). The highest value (52.30%) pertains to potentially endangered taxa, followed by critically endangered (26.23%), non-endangered (14.80), endangered (4.59%), vulnerable 1.18%, as well as extinct (0.16%) taxa.

**Figure 4.**
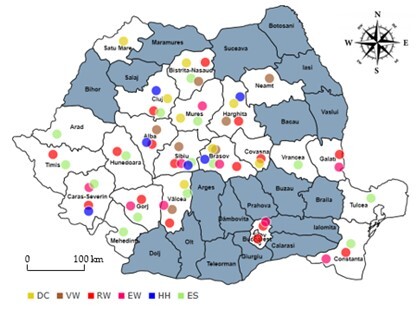
Geographical representation of Romania and the sites of collectors (red bullet - represents RW, blue bullet - represents HH, yellow bullet - represents DC, brown bullet - represents VW, green bullet - represents ES and pink bullet - represents EW).

By synthesising the data referring to the six lepidoptera collections (DC, EW, VW, RW, HH and ES), we identified 157 sites where the butterflies of the nymphalid family were collected between 1887 and 1984. The species were collected in all of Romania’s eight regions, from 23 counties and the capital of Bucharest, i.e. on 56.1% of Romania’s territory. I designed a map based on these data that reflects the distribution of species in eight regions whose counties are listed by collector: (1-DC), seven counties, BN, BV, CJ, CV, HR, MS, SB, all in the TR and SM regions, (2-EW), nine counties, BV, CS, CT, GL, GJ, IF, MS, VL, SB, from five of Romania’s regions: BT, DB, OT, MD si TR; (3-VW), seven counties, AB, BN, BV, HR, NT, VL, SB, from three regions: MD, OT, and TR; (4-RW), 16 counties, BN, BV, CJ, CS, CT, CV, GL, GJ, HR, HD, IF, MH, VL, SB, TM and Bucharest, from six of Romania’s regions: BT, DB, MD, MT, OT, TR and Bucharest; (5-HH), six counties, AB, BV, CS, CJ, HR, SB, from two of Romania’s regions: BT and TR; (6-ES), 16 counties, AB, AR, BN, BV, CS, CJ, CT, GJ, HD, MH, MS, VL, SB, TM, TL, VN, from six of Romania’s regions: BT, CR, DB, OT, MD, TR (Fig. 4).

**Figure 5.**
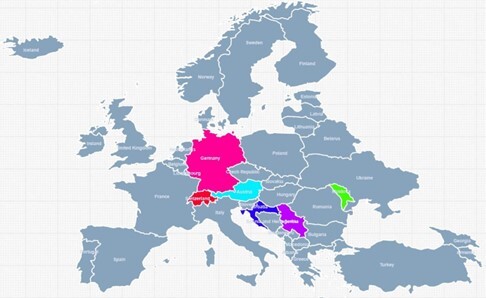
The species collected in other European countries are graphically represented.

The species collected in other European countries (Austria, Croatia, Switzerland, Germany, the Republic of Moldova and Serbia) were also graphically represented in Fig. 5.

**Conclusions**

Romania’s territory has a surface of 237,500 km^2^ that comprises a variety of geographic forms, from Alpine meadows to the Danube Delta’s hydrological profile. They are populated by a diverse fauna of lepidoptera containing over 200 diurnal butterfly species. Romania’s South, especially the South-East and South-West (BT and DB) is the region with the highest diversity of lepidoptera in the country (Szèkely 2008) and, at the same time, the northern limit of the distribution of their Balkan species in Europe (Ștefanescu et al. 2013, Wildman et al. 2022). The Danube’s gorge connects the Carpathian and Balkan Mountains. This geological formation has facilitated the entry of Balkan-type mountainous-subalpine elements into Romania, as far as the Retezat Mountains, for instance *Melitaearetyezatica* Diöszeghy, 1930. Given that the Carpathian Mountains do not exceed 2,500 m in height, their butterfly fauna is characterised mostly by subalpine, boreo-alpine and boreo-montane species (Szèkely 2008). Most endemic species have formed in the isolated, high regions of the Carpathian Mountains. They were analysed and included in the collections as follows: *Melitaearetyezatica* Diöszeghy, 1930, *Argynnispandoradacica* Hormuzaki, 1892 and *Boloriapales* ([Denis & Schiffermüller], 1775) *carpathomeridionalis* Crosson et Popescu-Gorj, 1963. The Romanian Catalogue of Lepidoptera records the presence of 90 species of which the Nymphalids are distributed as follows: BT (78 species), CR (60 species), TR (78 species), SM (66 species), OT (73 species), MT (77 species), MD (68 species) and DB (62 species) (Rakosy and Goia 2021). I have identified 49 species in the collections, which represent 54.44% of the species in Romania’s fauna. These species are distributed by counties as follows: BT with the counties (CS and TM); CR (AR); DB (CT and TL), MD (GL, NT, and VR); MT (IF); OT (GJ, MH, and VL); SM (SM), TR (AB, BN, BV, CV, CJ, HR, HD, MS, SB) and the capital Bucharest. Based on synthesising the collection data and on previously published data on species of this family (Stancă-Moise 2022), the present article presents 16 genera and 1966 specimens of the Nymphalid family collected in Romania since 137 years ago.

The article illustrates the biodiversity of the nymphalid family and their historical evolution in Romania and Europe, thus bringing an important historical, scientific and biogeographic "Council Directive (1992)" contribution to the study of butterflies. With the earliest data from 1887, my research complements current research on the presence, diversity and distribution of the nymphalid family in Romania until 1984. Alongside other rare, extinct or endangered species, the presence of nymphalid species with various endangerment levels in the patrimony of the Natural History Museum Sibiu enhances the scientific value of the museum’s collections and confirms their significance for ascertaining the biodiversity and distribution of the species throughout time. Future research will include digitising the Museum’s butterfly collection to facilitate the access to the collected specimens for specialists searching for historical data on the species.

## Supplementary Material

XML Treatment for Libythea celtis

XML Treatment for Argynnis paphia

XML Treatment for Argynnis paphia f. valensina

XML Treatment for Argynnis pandora

XML Treatment for Argynnis pandora dacica

XML Treatment for Argynnis (Speyeria) aglaja

XML Treatment for Argynnis adippe

XML Treatment for Argynnis (Fabriciana) niobef.cleodoxa

XML Treatment for Argynnis (Fabriciana) niobe niobe

XML Treatment for Argynnis (Fabriciana) niobevar.pelopia

XML Treatment for Argynnis laodice

XML Treatment for Argynnis latonia var. hungarica

XML Treatment for Issoria lathonia

XML Treatment for Brenthis ino

XML Treatment for Brenthis daphne

XML Treatment for Brenthis hecate

XML Treatment for Boloria euphrosyne

XML Treatment for Boloria selene

XML Treatment for Boloria dia

XML Treatment for Boloria pales carpathomeridionalis

XML Treatment for Inachis (Aglais) io

XML Treatment for Aglais urticae

XML Treatment for Polygonia c-album

XML Treatment for Polygonia egea

XML Treatment for Araschnia levana

XML Treatment for Araschnia levana f. porima

XML Treatment for Araschnia levana f. prorsa

XML Treatment for Araschnia levana f. intermedia

XML Treatment for Nymphalis antiopa

XML Treatment for Nymphalis polychloros

XML Treatment for Nymphalis xanthomelas

XML Treatment for Nymphalis vaualbum

XML Treatment for Euphydryas maturna

XML Treatment for Euphydryas orientalis

XML Treatment for Euphydryas aurinia

XML Treatment for Melitaea cinxia

XML Treatment for Melitaea phoebe

XML Treatment for Melitaea trivia

XML Treatment for Melitaea didyma

XML Treatment for Melitaea didyma alpina

XML Treatment for Melitaea didyma occidentalis

XML Treatment for Melitaea didyma meridionalis

XML Treatment for Melitaea didymoides

XML Treatment for Melitaea dictynna

XML Treatment for Melitaea aurelia

XML Treatment for Melitaea athalia

XML Treatment for Melitaea athalia mehadiensis

XML Treatment for Melitaea britomartis

XML Treatment for Melitaea diamina alpestris

XML Treatment for Melitaea nana

XML Treatment for Melitaea parthenoides

XML Treatment for Melitaea parthenie var. varia

XML Treatment for Melitaea retyezatica

XML Treatment for Melitaea asteria

XML Treatment for Melitaea athalia dictynnoides

XML Treatment for Limenitis populi

XML Treatment for Limenitis populi var. tremulae

XML Treatment for Limenitis camilla

XML Treatment for Limenitis sibilla (camilla)

XML Treatment for Limenitis reducta reducta

XML Treatment for Neptis aceris

XML Treatment for Neptis rivularis

XML Treatment for Neptis rivularis ludmila

XML Treatment for Neptis sappho

XML Treatment for Neptis lucilla

XML Treatment for Apatura ilia

XML Treatment for Apatura clytie

XML Treatment for Apatura ilia var. eos

XML Treatment for Apatura iris

XML Treatment for Apatura metis

## Figures and Tables

**Table 1.** Daniel DC’s collection.

Genus	Species	Collection period	Collection site	Legit	County	Region	Specimens	Status
*Argynnis* Fabricius, 1807	*Argynnispaphia* (Linnaeus, 1758)	1911-1930	Brașov, Cisnădioara, Hodod, Păltiniș, Porumbacu, Retezat Mountain, Sibiu	DC, Diószeghy, Kiss, Prall	BV, SM, SB, Southern Carpathians	SM, TR	16	NT
	*Argynnispandora* ([Denis & Schiffermüller], 1775)	no data	Cisnădioara, Porumbacu, Sibiu	DC, Prall	SB	TR	4	VU
	Argynnis (Speyeria) aglaja (Linnaeus, 1758)	1911-1930	Borsec, Colibița, Păltiniș	DC, Műller	BN, HR, SB	TR	7	LC
	*Argynnisadippe* ([Denis & Schiffermüller], 1775)	1906-1909	Bistrița, Brașov, Cisnădioara, Fedeleș, Hohod, Păltiniș, Mureș, Rodna, Sibiu	DC, Kiss	BN, BV, MS, SB	TR	14	NT
	Argynnis (Fabriciana) niobe (Linnaeus, 1758)	1904-1921	Borsec, Turnu Roșu	DC	HR, SB	TR	3	NT
	Argynnisniobeab.pelopia Borkhausen, 1788	no data	Sibiu	DC	SB	TR	1	NT
	*Argynnislaodice* (Pallas, 1771)	1908	Cibin, Hodod, Micăsasa	DC	SM, SB	SM, TR	4	EN
	*Argynniseris* Meigen, 1829	1921	Bazna	DC	SB	TR	5	EN
	Argynnislatoniaab.hungarica Aigner-Abafi, 1906	no data	Sibiu	DC	SB	TR	1	LC
*Issoria* Hűbner, 1819	*Issorialathonia* (Linnaeus, 1758)	1911-1934	Borsec, Sibiu, Retezat Mountain, Tălmaciu, Hodod	DC, Kiss	HR, SM, SB	SM, TR	6	LC
*Brenthis*, Hűbner, 1819	*Brenthisino* Rottemburg, 1775	1912-1928	Sfântu Gheorghe, Pleeshortalgy (?)	DC, Tiltsdur	CV	TR	2	VU
	*Brenthisdaphne* ([Denis & Schiffermüller], 1775)	no data	Hodod	Kiss	SM	SM	3	VU
*Boloria* Moore, 1900 (Clossiana), Moore, 1900	*Boloriaeuphrosyne* (Linnaeus, 1758)	1908-1911	Brașov, Fedeleș, Vâlcele, Borsec	DC, Deubel	BV, HR, Eastern Carpathians	TR	7	VU
	*Boloriaselene* ([Denis & Schiffermüller], 1775)	1887-1911		DC, Kiss	SB	TR	5	NT
	*Boloriadia* (Linnaeus, 1767)	1910-1922	Bazna, Sibiu, Șura Mică, Tălmaciu	DC, Kiss	SB	TR	6	LC
*Inachis* Hübner, 1819	Inachis (Aglais) io (Linnaeus, 1758)	no data	Păltiniș, Sibiu	DC, Kiss	SB	TR	4	LC
*Aglais* Dalman, 1816	*Aglaisurticae* (Linnaeus, 1758)	1891-1932	Fedeleș, Hodod, Sibiu, Retezat Mountain	DC, Kimakowics, Kiss	BV, SM, SB	SM, TR	12	NT
*Polygonia* Hübner, [1819]	*Polygoniac-album* (Linnaeus, 1758)	1908-1927	Hodod, Micăsasa, Sibiu, Retezat Mountain, Brașov	DC, Deubel, Dioszeghy, Kiss, Prall	SB, BV, SM, Southern Carpathians	SM, TR	16	NT
*Araschnia* Hübner, 1819	*Araschnialevana* (Linnaeus, 1758)	1922	Copșa Mică, Sibiu	DC	SB	TR	3	NT
	Araschnialevanaf.porima Ochsenheimer, 1807	1912	Sfântu Gheorghe	Miklos Tűtscher	CV	TR	1	NT
	Araschnialevanaf.prorsa Linnaeus, 1758	1908	Konzeshova (?)	DC	no data	no data	2	NT
	Araschnialevanaf.intermedia Stichel, 1906	1908	Brașov, Sibiu	DC	SB, BV	TR	3	NT
*Nymphalis* Kluk, 1802	*Nymphalisantiopa* (Linnaeus, 1758)	1909	Festebsch (?)	no data	no data	no data	2	VU
	*Nymphalispolychloros* (Linnaeus, 1758)	1887-1923	Sibiu	DC, Kiss, Borz3 N (?)	SB	TR	6	EN
	*Nymphalisxanthomelas* ([Denis & Schiffermüller], 1775)	no data	Păltiniș, Sibiu	DC	SB	TR	3	CR
	*Nymphalisvaualbum* ([Denis & Schiffermüller], 1775)	1912	Sibiu	DC	SB	TR	3	CR
*Euphydryas*, Scudder, 1872	*Euphydryasmaturna* (Linnaeus, 1758)	no data	Hodod, Sibiu	Deuber, Kiss	SM, SB	SM, TR	4	VU
	*Euphydryasaurinia* (Rottemburg, 1775)	no data	Sibiu, Șura Mică	DC	SB	TR	3	EN
*Melitaea* Fabricius, 1807	*Melitaeacinxia* (Linnaeus, 1758)	1907	Cluj, Hodod, Ocna Sibiului, Sibiu	DC, Kiss, Műller, Prall	CJ, SM, SB	SM, TR	8	NT
	*Melitaeaphoebe* ([Denis & Schiffermüller], 1775)	1911	Bistrița, Borsec, Sibiu, Ocna Sibiului	Alberti, DC, Kiss	BN, HR, SB	TR	9	NT
	*Melitaeatrivia* ([Denis & Schiffermüller], 1775)	no data	Ocna Sibiului, Sâmbăta, Sibiu	DC, Kiss	BV, SB	TR	4	NT
	*Melitaeadidyma* (Esper, 1778)	1909-1912	Bâlea, Cluj-Napoca, Hodod, Rășinari, Brașov, Sibiu	DC, Kiss, Műller	BV, CJ, SM, SB	SM, TR	17	LC
	*Melitaeadictynna* Esper, 1778	1901-1928	Sibiu, Plecska (?)	DC	SB	TR	4	VU
	*Melitaeaaurelia* Nickerl, 1850	1911-1930	Bazna, Borsec, Brașov, Ocna Sibiului, Sibiu	DC	BV, HR, SB	TR	12	VU
	*Melitaeaathalia* (Rottemburg, 1775)	1904-1934	Bâlea, Brașov, Gușterița, Hodod, Sibiu	DC, Kiss	BV, SM, SB	SM, TR	26	NT
	*Melitaeadiaminaalpestris* Fruhstorfer, 1917	no data	Hodod, Arpaș	DC, Kiss	SM, SB	SM, TR	3	NT
	*Melitaeanana* Rehfons, 1910 sin. *Euphydryasaurinia* (Rottemburg, 1775)	no data	Lands (?), Sibiu	DC	SB	TR	2	VU
	*Melitaeaparthenie* Godart, 1822 sin. *Melitaeaparthenoides* Keferstein, 1851	no data	Hodod	Kiss	SM	SM	1	VU
	*Melitaeaathalia* (Rottemburg, 1775) f. *Melitaearetyezatica* Diöszeghy, 1930	1919-1922	Retezat Mountain	Diöszeghy	Southern Carpathians	TR	9	Endemic
	*Melitaeaathaliadictynnoides* Hormuzaki, 1898	no data	Sibiu	DC	SB	TR	4	NT
*Limenitis* Fabricius, 1807	*Limenitispopuli*, (Linnaeus, 1758)	no data	Păltiniș	DC	SB	TR	2	VU
	Limenitisab.tremulae Esper, 1800	1922	Păltiniș, Retezat Mountain	DC	SB, Southern Carpathians	TR	2	VU
	*Limenitiscamilla* Linnaeus, 1764	1915	Păltiniș, Cheile Turzii	DC, Műller	SB, CJ	TR	2	VU
*Neptis* Fabricius, 1807	*Neptisaceris* sensu Lhomme, 1924	1911-1929	Bran, Șura Mare, Retezat Mountain, Sibiu, Tălmaciu, Hodod	DC, Diöszeghy, Kiss	BV, SM, SB, Southern Carpathians	SM, TR	10	VU
	*Neptislucilla* ([Denis & Schiffermüller], 1775) sin. *Neptisrivularis* (Scopoli, 1763)	1915-1924	Borsec, Păltiniș, Slimnic	DC, Műller	HR, SB	TR	4	NT
*Apatura* Fabricius, 1807	*Apaturailia* ([Denis & Schiffermüller], 1775)	1928-1929	Greliesei (?), Cluj	Peterfi	CJ	TR	2	VU
	*Apatura* v. *clytie* ([Denis & Schiffermüller], 1775)	1909-1933	Gușterița, Hodod, Sibiu	DC, Kiss	SM, SB	SM, TR	4	VU
	*Apatura* ab. *eos rossi* Rossi, 1794	no data	Sibiu	DC	SB	TR	2	VU
	*Apaturairis* (Linnaeus, 1758)	1925-1926	Păltiniș, Hodod, Râu Sadului, Sibiu	DC, Kiss	SM, SB	SM, TR	5	VU

**Table 2.** Eugen Worell’s collection.

Genus	Species	Collection period	Collection site	Legit	County	Region	Specimens	Status
*Argynnis* Fabricius, 1807	*Argynnispaphia* (Linnaeus, 1758)	1932-1957	Carinthia Plocken, Chișinău, Sibiu, Lintz	Kolar EW	SB, Chișinău, Tirol	TR, RMO, AU	4	NT
	Argynnispaphiaf.valensina [Esper, 1798]	1932-1953	Carinthia, Cozia, Eforie Sud, Ocna Sibiului, Sibiu	EW Kolar	CT, SB	DB, TR, AU	23	NT
	*Argynnispandora* ([Denis & Schiffermüller], 1775)	1948	Cozia Montain	EW	VL	OT	7	VU
	*Argynnispandoradacica* Hormuzaki, 1892	1930-1931	Tecuci, Retezat Mountain	Alexinschi Dioszegy	GL, Southern Carpathians	MD	3	VU
	Argynnis (Speyeria) aglaja (Linnaeus, 1758)	1935-1953	Băile Herculane, Păltiniș, Sibiu	EW	CS, SB	BT, TR	13	LC
	Argynnis (Fabriciana) adippe ([Denis & Schiffermüller], 1775)	1933-1952	Brașov, Cisnădioara, Măgura Cisnădiei, Gușterița, Sibiu	EW	BV, SB	TR	18	NT
	*Argynnisniobe* (Linnaeus, 1758)	1921-1948	Bazna, Păltiniș Sibiu, Salsburg, Schlenken	EW	SB	TR, AU	12	NT
	*Argynnislaodice* (Pallas, 1771)	1938-1953	Sibiu	EW	SB	TR	17	EN
*Inachis* Hübner, 1819	Inachis (Aglais) io (Linnaeus, 1758)	1936-1953	Chișinău	EW	Chișinău	RMO	11	LC
*Aglais* Dalman, 1816	*Aglaisurticae* (Linnaeus, 1758)	1937	Chișinău	EW	Chișinău	RMO	13	NT
*Polygonia* Hübner, [1819]	*Polygoniac-album* (Linnaeus, 1758)	1936-1952	Chișinău, Ocna Sibiului, Sibiu	EW	SB, Chișinău	RMO	17	NT
	*Polygoniaegea* (Cramer, 1775)	1910	Pola Istrien	EW	-	HR	3	EX
*Araschnia* Hübner, 1819	*Araschnialevana* (Linnaeus, 1758)	1930	Salisberg	EW	-	GE	2	NT
	*Araschniaprorsa* Linnaeus, 1758	1924-1957	Sibiu, Sighișoara	EW	SB, MS	TR	11	NT
*Nymphalis* Kluk, 1802	*Nymphalisantiopa* (Linnaeus, 1758)	1934-1955	Cisnădioara, Sibiu, Turnișor Sibiu	EW	SB	TR	14	VU
	*Nymphalispolychloros* (Linnaeus, 1758)	1888-1953	Sibiu	Umgebung EW	SB	TR	13	EN
	*Nymphalisxanthomelas* ([Denis & Schiffermüller], 1775)	1939	Sibiu	EW	SB	TR	3	CR
*Euphydryas*, Scudder, 1872	*Euphydryasmaturna* (Linnaeus, 1758)	1935-1942	Băile Herculane	EW	CS	BT	5	VU
	*Euphydryasaurinia* (Rottemburg, 1775)	1905	Râșnov	EW	BV	TR	3	EN
*Melitaea* Fabricius, 1807	*Melitaeacinxia* (Linnaeus, 1758)	1936-1983	Sibiu, Băile Herculane, Cernica	EW	CS, IF, SB	BT, MT, TR	15	NT
	*Melitaeaphoebe* ([Denis & Schiffermüller], 1775)	1931-1939	Eforie Sud, Techirgiol, Sighișoara, Chișinău, Sibiu, Gușterița	EW	Chișinău, CT, MS, SB	DB, TR, RMO	11	NT
	*Melitaeatrivia* ([Denis & Schiffermüller], 1775)	1935-1954	Gușterița, Sibiu, Chișinău	EW	SB, Chișinău	TR, RMO	34	NT
	*Melitaeadidyma* (Esper, 1778)	1938-1939	Gușterița, Sibiu	EW	SB	TR	2	LC
	*Melitaeadidymaalpina* Staudinger, 1861	1938-1957	Sibiu	EW	SB	TR	10	VU
	*Melitaeadidymameridionalis* Staudinger, 1870	1945-1953	Sibiu	EW	SB	TR	10	VU
	*Melitaeaaurelia* Nieckerl, 1850	1920-1956	Sibiu, Sighișoara	DC, EW	SB, MS	TR	7	VU
	*Melitaeaathalia* (Rottemburg, 1775)	1927-1953	Bularda, Păltiniș, Sibiu	EW Alexinschi	SB, Chișinău	TR, RMO	24	NT
	*Melitaeaathaliamehadiensis* Gerhard, 1822	1938-1942	Băile Herculane	EW	CS	BT	4	NT
	*Melitaeadidymoides* Eversmann, 1847 sin. *Melitaeadidyma* (Esper, 1778)	1934-1939	Buciumeni, Bularda	EW	GL, Chișinău	RMO, MD	4	VU
	*Melitaeadictynna* Esper, 1778 sin. *Melitaeadiamina* (Lang, 1789)	1930-1956	Sibiu, Sighișoara, Tismana	EW	GJ, MS, SB	OT TR	10	VU
	*Melitaeaparthenie* Godart, 1822 sin. *Melitaeaparthenoides* Keferstein, 1851	1926-1953	Sibiu, Kaiserstuhl (?)	EW	SB	TR	2	VU
	Melitaeaparthenievar.varia Meyer-Dűr, 1851	1927-1930	Tirol Rotmoostal, Tirol Gaisbergatal	Holik	Tirol	AU	2	VU
	*Melitaeaasteria* Freyer, 1828	1938-1958	Sibiu	EW	SB	TR	9	VU
*Limenitis* Fabricius, 1807	*Limenitispopuli*, (Linnaeus, 1958)	1950	Sibiu	EW	SB	TR	2	VU
	Limenitisab.tremulae Esper, 1800	1962	Băile Herculane	EW	CS	BT	2	VU
	*Limenitiscamilla* Linnaeus, 1764	1905-1939	Băile Herculane, Sibiu, Pontresina	EW	CS	BT, TR CH	7	VU
*Neptis* Fabricius, 1807	*Neptisrivularis* (Scopoli, 1763)	1936	Băile Herculane	EW	CS	BT	6	NT
	*Neptislucilla* ([Denis & Schiffermüller], 1775)	1936-1952	Băile Herculane, Sibiu	EW	CS	BT, TR	26	NT
*Apatura* Fabricius, 1807	*Apaturametis* Freyer, 1829	no data	no data	no data	no data	no data	2	VU
	*Apaturailia* ([Denis & Schiffermüller], 1775)	1939-1959	Sibiu	EW	SB	TR	6	VU
	*Apaturailiaeos* Rossi, 1794	1938	Sibiu	EW	SB	TR	2	VU
	*Apaturairis* (Linnaeus, 1958)	1927-1956	Palamea, Sibiu, Șercaia	EW	BV, SB	TR	5	VU

**Table 3.** Viktor Veindel’s collection.

Genus	Species	Collection period	Collection site	Legit	County	Region	Specimens	Status
*Argynnis* Fabricius, 1807	*Argynnispaphia* (Linnaeus, 1758)	1903-1956	Cisnădie, Borsec, Cheile Bicazului, Ghiorghieni, Râu Vadului	VW	SB, HR, NT	TR	17	NT
	*Argynnispandora* ([Denis & Schiffermüller], 1775)	1904-1955-	Cisnădioara, Sibiu, Râu Vadului, Cozia	VW	SB, VL	OT, TR	4	VU
	Argynnis (Speyeria) aglaja (Linnaeus, 1758)	1903-1954	Borsec, Cisnădioara, Colibița, Gușterița, Sibiu	VW	BN, HR, SB	TR	14	LC
	Argynnis (Fabriciana) adippe ([Denis & Schiffermüller], 1775)	1903-1956	Cisnădie, Gușterița, Sibiu	VW	SB	TR	7	NT
	*Argynnisadippe f. cleodoxa* Ochsenheimer, 1816	1906-1956	Cisnădie, Gușterița, Sibiu	VW	SB	TR	8	NT
	Argynnis (Fabriciana) niobe (Linnaeus, 1758)	1904-1922	Cârțișoara, Sibiu	VW	SB	TR	4	NT
	*Argynnislaodice* (Pallas, 1771)	1907-1921	Cisnădioara, Gușterița, Saschiz	VW	SB	TR	3	EN
*Issoria* Hűbner, 1819	*Issorialathonia* (Linnaeus, 1758)	1903-1958	Gușterița, Borsec, Cisnădie, Păltiniș, Cozia, Paltin, Râu Vadului	VW	SB	TR	14	LC
*Brenthis* Hűbner, 1819	*Brenthisino* Rottemburg, 1775	1921	Borsec	VW	HR	TR	4	VU
	*Brenthisdaphne* ([Denis & Schiffermüller], 1775)	1954-1956	Păltiniș, Râu Vadului	VW	SB	TR	2	VU
	*Brenthishecate* ([Denis & Schiffermüller], 1775)	1920-1956	Gușterița, Saschiz	VW	SB	TR	6	VU
*Boloria* Moore, 1900	*Boloriaeuphrosyne* (Linnaeus, 1758)	1904-1956	Cisnădie, Șura Mică, Sibiu	VW	SB	TR	10	VU
	*Boloriaselene* ([Denis & Schiffermüller], 1775)	1903-1956	Cisnădioara, Gușterița, Șura Mică	VW	SB	TR	12	NT
	*Boloriadia* (Linnaeus, 1767)	1904-1958	Borsec, Cisnădioara, Gușterița, Păltiniș, Șura Mică, Râu Vadului	VW	SB	TR	23	LC
	*Boloriapales* ([Denis & Schiffermüller], 1775) *carpathomeridionalis* Crosson et Popescu-Gorj, 1963	1957	Suru	VW	Făgăraș Mountain	TR	5	VU Endemic
*Inachis* Hübner, 1819	Inachis (Aglais) io (Linnaeus, 1758)	1904-1952	Cisnădie, Sibiu	VW	SB	TR	14	LC
*Aglais* Dalman, 1816	*Aglaisurticae* (Linnaeus, 1758)	1903-1930	Gușterița, Păltiniș, Sibiu	VW	SB	TR	10	NT
*Polygonia* Hübner, [1819]	*Polygoniac-album* (Linnaeus, 1758)	1904-1956	Borsec, Cisnădie, Cisnădioara, Cozia, Gușterița, Păltiniș, Sadu, Râu Vadului	VW	HR, VL, SB	OT, TR	20	NT
*Araschnia* Hübner, 1819	*Araschnialevana* (Linnaeus, 1758)	-	Sibiu	VW	SB	TR	2	NT
	Araschnialevanaf.prorsa (Linnaeus, 1758)	1903-1958	Borsec, Colibița, Ghiorghieni, Păltiniș, Sibiu, Râu Sadului	VW	BN, HR, SB	TR	14	NT
*Nymphalis* Kluk, 1802	*Nymphalisantiopa* (Linnaeus, 1758)	1905-1955	Cisnădioara, Gușterița, Păltiniș, Sibiu	VW	VL, SB	OT, TR	24	VU
	*Nymphalispolychloros* (Linnaeus, 1758)	1903-1952	Gușterița, Păltiniș, Sibiu	VW	SB	TR	10	EN
	*Nymphalisxanthomelas* ([Denis & Schiffermüller], 1775)	1904	Sibiu	VW	SB	TR	2	CR
	*Nymphalisvaualbum* ([Denis & Schiffermüller], 1775)	1908-1922	Cisnădie, Păltiniș	VW	SB	TR	2	CR
*Euphydryas* Scudder, 1872	*Euphydryasaurinia* (Rottemburg, 1775)	1920	Saschiz	VW	SB	TR	2	EN
*Melitaea* Fabricius, 1807	*Melitaeacinxia* (Linnaeus, 1758)	1956	Șura Mică, Târnăvioara	VW	SB	TR	5	NT
	*Melitaeaphoebe* ([Denis & Schiffermüller], 1775)	1903-1956	Gușterița, Sebeș, Sibiu	VW	AB, SB	TR	8	NT
	*Melitaeatrivia* ([Denis & Schiffermüller], 1775)	1920-1958	Gușterița, Prislop, Tălmaciu, Târnăvioara	VW	SB	TR	14	NT
	*Melitaeadidyma* (Esper, 1778)	1903-1956	Cisnădioara, Cisnădie, Gușterița, Paltin, Turnu Roșu, Râul Vadului, Tâmpa	DC VW	BV, SB	TR	33	LC
	*Melitaeaathalia* (Rottemburg, 1775)	1903-1956	Borsec, Cisnădie, Cisnădioara, Colibița, Cozia, Cheile Bicazului, Șura Mică	DC VW	BN, HR, NT, SB, VL	MD, OT, TR, RS	37	NT
	*Melitaeaaurelia* Nickerl, 1850	1907-1955	Gușterița, Viile Sibiului, Polja	DC VW	SB	TR, RS	13	VU
*Limenitis* Fabricius, 1807	*Limenitispopuli*, (Linnaeus, 1958)	1904-1954	Cheile Bicazului, Sibiu	VW	HR, NT, SB	TR	5	VU
	Limenitispopulif.tremulae Esper, 1798	no data	no data	no data	no data	no data	1	VU
	*Limenitiscamilla* (Linnaeus, 1964)	1954	Cheile Bicazului, Colibița	VW	BN, HR, NT	TR	7	VU
*Neptis* Fabricius, 1807	*Neptisrivularis* (Scopoli, 1763)	1904-1957	Făgăraș, Sibiu	VW	BV, SB	TR	6	NT
	*Neptisrivularisludmila* Nordmann, 1851	1921-1954	Borsec, Tușnad	VW	HR, NT	TR	3	NT
	*Neptissappho* Pallas, 1771	1907-1956	Gușterița, Șura Mică, Râu Vadului	VW	SB	TR	6	VU
*Apatura* Fabricius, 1807	*Apaturailia* ([Denis & Schiffermüller], 1775)	1904	Sibiu	VW	SB	TR	2	VU
	*Apaturaclytie* ([Denis & Schiffermüller], 1775)	1904-1905	Cisnădioara, Sibiu	VW	SB	TR	3	VU
	*Apaturairis* (Linnaeus, 1958)	1904-1954	Cisnădie, Colibița, Gheorghieni, Păltiniș, Sibiu	VW	BN, HR, SB	TR	9	VU

**Table 4.** Heinrich Hann von Hannenheim’s collection.

Genus	Species	Collection period	Collection site	Legit	County	Region	Specimens	Status
*Issoria* Hübner, 1819	*Issorialathonia* (Linnaeus, 1758)	1955-1963	Apoldul de Sus, Bărcaciu, Cisnădioara, Tălmaciu, Sibiu, Șopa	HH	SB	TR	6	LC
*Brenthis* Hűbner, 1819	*Brenthishecate* ([Denis & Schiffermüller], 1775)	1928-1956	Galeș, Gușterița, Cluj, Sibiu	HH	CJ, SB	TR	5	VU
*Boloria* Moore, 1900	*Boloriaeuphrosyne* (Linnaeus, 1758)	1954-1964	Gușterița, Băile Herculane, Sibiu, Șopa	HH	CS, SB	BT, TR	6	VU
	*Boloriaselene* ([Denis & Schiffermüller], 1775) sin. *Boloriaselenis* (Eversmann, 1837)	1954-1964	Bungard, Gușterița, Sibiu	HH	SB	TR	8	NT
	*Boloriadia* (Linnaeus, 1767)	1954-1963	Gușterița, Obreja, Slimnic, Sibiu, Tălmaciu	HH	SB	TR	10	LC
	*Boloriapales* ([Denis & Schiffermüller], 1775) carprathomeridionalis Crosson et Popescu-Gorj, 1963	1963	Chica Pietrilor	HH	BV	TR	3	VU
*Inachis* Hübner, 1819	Inachis (Aglais) io (Linnaeus, 1758)	1955-1957	Păltiniș, Sâmbata, Sibiu	HH	BV, SB	TR	4	LC
*Polygonia* Hübner, [1819]	*Polygoniac-album* (Linnaeus, 1758)	1954-1957	Gușterița, Păltiniș, Sebeș, Sibiu, Tălmaciu	HH	AB, SB	DB, TR	13	NT
*Araschnia* Hübner, 1819	*Araschnialevana* (Linnaeus, 1758)	1954-1964	Gușterița, Slimnic, Sebeș, Sibiu	HH	AB, SB	TR	9	NT
*Nymphalis* Kluk, 1802	*Nymphalisantiopa* (Linnaeus, 1758)	1954-1955	Păltiniș	HH	SB	TR	3	VU
*Melitaea* Fabricius, 1807	*Melitaeacinxia* (Linnaeus, 1758)	1955-1957	Cisnădioara, Gușterița, Slimnic	HH	SB	TR	3	NT
	*Melitaeadidyma* (Esper, 1778)	1955-1962	Apoldul de Sus, Sibiu, Tălmaciu	HH	SB	TR	8	LC
	*Melitaeaaurelia* Nickerl, 1850	1956-1965	Ocna Sibiului, Gușterița	HH	SB	TR	6	VU
	*Melitaeaathalia* (Rottemburg, 1775)	1954-1958	Apoldul de Sus, Gușterița, Sibiu, Tălmaciu	HH	SB	TR	10	NT
*Limenitis* Fabricius, 1807	*Limenitispopuli* (Linnaeus, 1958)	1963-1964	Sibiu	HH	SB	TR	3	VU
*Neptis* Fabricius, 1807	*Neptisaceris* sensu Lhomme, 1924	1956-1958	Sibiu, Tălmaciu	HH	SB	TR	4	VU
	*Neptislucilla* ([Denis & Schiffermüller], 1775)	1954-1964	Sibiu, Sâmbata	HH	BV, SB	TR	5	NT
*Apatura* Fabricius, 1807	*Apaturailia* ([Denis & Schiffermüller], 1775)	1954-1964	Prejmer, Sâmbata, Tușnad	HH	BV, HR, SB	TR	3	VU
	*Apaturairis* (Linnaeus, 1958)	1954-1964	Măgura Cisnădiei, Păltiniș, Sâmbata, Tușnad	HH	BV, HR, SB	TR	8	VU

**Table 5.** Rolf Weihrauch’s collection.

Genus	Species	Collection period	Collection site	Legit	County	Region	Specimens	Status
*Argynnis* Fabricius, 1807	*Argynnispandora* ([Denis & Schiffermüller], 1775)	1965-1978	Băile Herculane, Băneasa, Canaraua Fetii, Cozia, Hagieni, Iormac, Sibiu	RW	CS, CT, VL, B, SB	DB, OT, TR	15	VU
*Inachis* Hübner, 1819	Inachis (Aglais) io (Linnaeus, 1758)	1955-1971	Colibița, Cozia, Iormac, Praid, Sibiu, Vlădeasa	RW	BN, CT, HR, SB, VL	DB, OT, TR	9	LC
*Aglais* Dalman, 1816	*Aglaisurticae* (Linnaeus, 1758)	1954-1977	Băile Herculane, Păltiniș, Someș, Tușnad, Vlădeasa	RW	CS, HR, SB	BT, TR, Western Carpathians	8	NT
*Polygonia* Hübner, [1819]	*Polygonia c-аlbum* (Linnaeus, 1758)	1954-1977	Covasna, Gârbova, Domogled, Păltiniș, Sibiu	RW	CS, CT, CV, GL, SB	DB, OT, MD, TR	16	NT
*Araschnia* Hübner, 1819	*Araschnialevana* (Linnaeus, 1758)	1954-1975	Băile Herculane, Covasna, Gușterița, Lugoj, Porumbacu, Tușnad, Negoi, Sibiu	RW	CS, CV, HR, SB, TM	BT, TR	31	NT
*Nymphalis* Kluk, 1802	*Nymphalisantiopa* (Linnaeus, 1758)	1951-1970	Colibița, Cozia, Covasna, Lotru, Păltiniș	RW	BN, CV, HR, VL, SB	DB, OT, TR	10	VU
	*Nymphalispolychloros* (Linnaeus, 1758)	1954-1971	Canaraua Fetii, Sibiu	RW	CT, SB	DB, TR	7	EN
	*Nymphalisxanthomelas* ([Denis & Schiffermüller], 1775)	1956	Domogled	RW	CS	BT	4	CR
	*Nymphalisvaualbum* ([Denis & Schiffermüller], 1775)	1942-1970	Căprioara, Poiana Mărului	Pelits, RW, Teleki	BV, CS	BT, TR	3	CR
*Euphydryas*, Scudder, 1872	*Euphydryasorientalis* (Herrich-Schaffer, [1851]) sin. *Euphydryasaurinia* (Rottemburg, 1775)	1922	Battnlta (?)	no data	no data	no data	2	CR
*Melitaea* Fabricius, 1807	*Melitaeacinxia* (Linnaeus, 1758)	1954-1975	Băile Herculane, Canaraua Fetii, Domogled, Găneasa, Retezat Mountain	RW	CS, CT, IF, Southern Carpathians	DB, MT	15	NT
	*Melitaeaphoebe* ([Denis & Schiffermüller], 1775)	1954-1978	Băile Herculane, Domogled, Hagieni, Galați	RW	CS, CT, GL, MH, GJ	BT, DB, OT, MD	24	NT
	*Melitaeadidyma* Esper, 1778	1954-1971	Băile Herculane, Domogled, Gușterița, Retezat Mountain, Sibiu, Tușnad	RW	CS, MH, GJ, SB	BT, OT, TR	18	LC
	*Melitaeadidymaoccidentalis* Staudinger, 1861	1943-1962	UlrichSBerg Kärnten/Karintia	Thurner	-	AU	2	VU
	*Melitaeadictynna* Esper, 1778 sin. *Melitaeadiamina* (Lang, 1789)]	1955-1977	Sadu, Someșu Rece	RW	CJ, SB	TR	4	VU
	*Melitaeaaurelia* Nickerl, 1850	1952-1963	Băile Herculane	RW	CS	BT	5	VU
	*Melitaeabritomartis* Assmann, 1847	1956-1974	Băile Herculane	RW	CS	BT	6	NT
	*Melitaeaathalia* (Rottemburg, 1775)	1950-1977	Avrig, Băile Herculane, Buila Vânturarița, Hagieni, Praid, Tușnad, Valea Vinului, Gioagiu, Someșul Rece	RW	BN, CS, CJ, CT, HR, HD, VL	BT, DB, OT, TR	12	NT
	*Melitaeanevadensis* Oberthür, 1904	1956-1967	Băile Herculane, Domogled	RW	CS, MH, GJ	BT, OT	15	NT
*Limenitis* Fabricius, 1807	*Limenitispopuli* (Linnaeus, 1958)	1954-1964	Ceahlău, Hoghilag, Obreja, Tușnad	RW	CS, HR, SB	BT, TR, Eastern Carpathians	4	VU
	Limenitispopulif.tremulae Esper, 1798	1964-1976	Băile Herculane, Domogled	RW	CS, MH, GJ	BT, OT	4	VU
	*Limenitiscamilla* (Linnaeus, 1764)	1954-1967	Domogle, Tușnad	RW	CS, HR, GJ, MH	BT, OT, TR	7	VU
	*Limenitisreducta* Staudinger, 1901	1961-1971	Domogled, Miercurea Băi	RW	CS, MH, GJ, SB	BT, OT, TR	4	EN
*Neptis* Fabricius, 1807	*Neptissappho* [Pallas, 1771]	1963-1969	Băile Herculane, Domogled, Sibiu	RW	CS, MH, GJ, SB	BT, OT, TR	6	VU
	*Neptisrivularis* (Scopoli, 1763)	1954-1974	Gușterița, Domogled, Someș	RW	CS, CJ, GJ, MH, SB	BT, OT, TR	9	NT
	*Neptislucilla* ([Denis & Schiffermüller], 1775) syn. *Neptisrivularis* (Scopoli, 1763)]	1962-1974	Gușterița, Domogled, Someș	RW	CS, CJ, GJ, MH, SB	BT, OT, TR	9	NT
*Apatura* Fabricius, 1807	*Apaturailia* ([Denis & Schiffermüller], 1775)	1971	Canaraua Fetii	RW	CT	DB	1	VU
	*Apaturaclytie* ([Denis & Schiffermüller], 1775)	1953-1963	Băile Herculane, Tușnad	RW	CS, HR	BT, TR	8	VU

**Table 6.** Eckbert Schneider’s collection.

Genus	Species	Collection period	Collection site	Legit	County	Region	Specimens	Status
*Libythea* Fabricius, 1807	*Libytheaceltis* (Laicharting, 1782)	1949	Băile Herculane	ES	CS	BT	1	EN
*Argynnis* Fabricius, 1807	*Argynnispaphia* (Linnaeus, 1758)	1952-1978	Agnita, Lotrioara, Laita, Hamba, Mailat, Sighișoara, Sibiel, Sibiu, Zarand	ES	AR, BV, MS, SB, Western Carpathians	BT, CR, TR	13	NT
	*Argynnispandora* ([Denis & Schiffermüller], 1775)	1954- 1979	Bestepe, Bruiu, Măcin, Hagieni, CA Rosetti, Sibiu	ES	CT, SB, TL	DB, TR	8	VU
	Argynnis (Speyeria) aglaja (Linnaeus, 1758)	1973-1982	Hamba, Șura Mare, Cerna, Laita, Porumbacu	ES	BV, CS, MH, GJ, SB	BT, OT, TR	7	LC
	Argynnis (Fabriciana) niobe, (Linnaeus, 1758)	1952-1984	Agnita, Cisnădie, Gușterița, Tomnatic, Beja, Șura Mare, Slimnic, Sighișoara, Tălmaciu, Zarand	ES	SB, HD, MS, TM, Western Carpathians	BT, TR	16	NT
*Issoria* Hűbner, 1819	*Issorialathonia* (Linnaeus, 1758)	1955-1967	Iortmac, Slimnic, Șiria, Sibiu, Turda	ES	AR, CJ, CT, SB	CR, DB, TR	12	LC
*Brenthis* Hűbner, 1819	*Brentisdaphne* ([Denis & Schiffermüller], 1775)	1956-1960	Sibiu, Zarandului Mountain	ES	Western Carpathians	TR	8	VU
	*Brentishecate* ([Denis & Schiffermüller], 1775)	1949-1976	Agărbiciu, Buia, Bruiu, Nocrich, Făget, Cincu, Gușterița, Șeica Mare, Șura Mare, Târnava	ES	CJ, SB	TR	16	VU
*Boloria* Moore, 1900	*Boloriaeuphrosyne* (Linnaeus, 1758)	1955-1969	Cozia, Rodna	ES	BN, VL, Eastern Carpathians	OT, TR	4	VU
	*Boloriaselene* ([Denis & Schiffermüller], 1775)	1955-1984	Axente Sever, Bruiu, Cincu, Cisnadie, Copșa Mică, Hamba, Podu Olt, Porumbacu, Nocrich, Chiș-Chiș, Șura Mare, Slimnic, Rusciori, Porumbacu	ES	BV, SB	TR	27	NT
	*Boloriadia* (Linnaeus, 1767)	1952-1984	Agârbiciu, Avrig, Cincu, Clopotiva, Cluj Napoca, Copșa Mică, Gușterița, Făget, Hagieni, Podu Olt, Râu Vadului, Slimnic, Sibiu, Șiria, Șura Mare, Veseud	ES	AR, CJ, CT, HD, VL, SB,	CR, DB, OT, TR	36	LC
*Inachis* Hübner, 1819	Inachis (Aglais) io (Linnaeus, 1758)	1960-1976	Gușterința, Zarand	ES	SB, Western Carpathians	TR	2	LC
*Aglais* Dalman, 1816	*Aglaisurticae* (Linnaeus, 1758)	1964-1974	Hamba, Sibiu, Vrancea	ES	SB, VN	MD, TR	4	NT
*Polygonia* Hübner, [1819]	*Polygoniac-album* (Linnaeus, 1758)	1963-1975	Brașov, Cincu, Cisnădie, Gușterița, Hamba, Rusciori	ES	BV, SB	TR	7	NT
*Araschnia* Hübner, 1819	*Araschnialevana* (Linnaeus, 1758)	1967-1981	Făgăraș, Gușterița, Podu Olt, Sighișoara, Slimnic, Veștem	ES	BV, MS, SB	TR	11	NT
*Nymphalis* Kluk, 1802	*Nymphalisantiopa* (Linnaeus, 1758)	1953	Sibiu	ES	SB	TR	2	VU
	*Nymphalispolychloros* (Linnaeus, 1758)	1953-1983	Cașolț, Cornățel, Sibiu	ES	SB	TR	3	EN
Euphydryas Scudder, 1872	*Euphydryasmaturna* (Linnaeus, 1758)	1960	Chiș-Chiș	ES			4	VU
	*Euphydryasaurinia* (Rottemburg, 1775)	1960-1982	Gherdeal, Șercaia	ES	BV, VL	OT, TR	5	EN
*Melitaea* Fabricius, 1807	*Melitaeacinxia* (Linnaeus, 1758)	1954-1983	Cincu, Cornățel, Gușterița, Nocrich, Făget, Roșia, Șiria, Șura Mare, Slimnic, Sibiu, Saschiz	ES	AR, CJ, MS, SB	CR, TR	29	NT
	*Melitaeaphoebe* ([Denis & Schiffermüller], 1775)	1952-1975	Axente Sever, Sibiu, Viile Sibiului, Nocrich, Făget, Gușterița, Hagieni, Hamba, Clopotiva, Chiș-Chiș, Cincu, Merghindeal, Podu Olt, Zarand	ES	CT, SB	DB, TR, Southern Carpathians	44	NT
	*Melitaeatrivia* ([Denis & Schiffermüller], 1775)	1970-1976	Axente Sever, Băile Herculane, Gușterița, Hagieni, Nocrich, Orlat, Sibiel, Sibiu, Slimnic, Șura Mare, Veștem	ES	CS, CT, SB	BT, DB, TR	20	NT
	*Melitaeadidyma* (Esper, 1778)	1952-1980	Agnita, Băile Herculane, Cenade, Cornățel, Făget, Gușterița, Hamba, Lotrioara, Orlat, Orșova, Podu Olt, Șiria, Slimnic, Șeica Mare, Șura Mare, Veștem	ES	AB, AR, CS, CJ, MH, VL, SB	BT, CR, OT, TR	55	NT
	*Melitaeaaurelia* Nickerl, 1850	1954-1984	Aiud, Axente Sever, Cenade, Clopotiva, Bruiu, Gușterița, Fărget, Moșna, Râu Vadului, Roșia, Șiria, Scărița Belioara, Nocrich, Șura Mare, Șeica Mare, Șercaia	ES	AB, AR, CJ, BV, HD, VL, SB, Western Carpathians	CR, OT, TR	55	VU
	*Melitaeaathalia* (Rottemburg, 1775)	1954-1982	Băile Herculane, Cincu, Chiș-Chiș, Feldioara, Fărget, Gusu, Gușterița, Hamba, Mohu, Nocrich, Poplaca, Șercaia	ES	BV, CS, CJ, SB	BT, TR	23	NT
*Neptis* Fabricius, 1807	*Neptisrivularis* (Scopoli, 1763)	1974-1982	Arpășel, Făgăraș, Sibiu	ES	BV, SB	TR	8	NT
	*Neptislucilla* ([Denis & Schiffermüller], 1775)	1958-1974	Bejan, Gușterița, Șiria	ES	AR, HD, SB	CR TR	8	NT
	*Neptissappho* Pallas, 1771	1970-1975	Bejan, Șeica Mare, Veștem	ES	HD, SB	TR	6	VU
*Apatura* Fabricius, 1807	*Apaturailia* ([Denis & Schiffermüller], 1775)	1968-1970	Sibiu, Șeica Mare	ES	SB	TR	2	VU
	*Apaturairis* (Linnaeus, 1958)	1959-1982	Băile Herculane, Păltiniș, Șeica Mare	ES	CS, SB	BT, TR	4	VU

## References

[B8257105] Burnaz S. (2002). Fauna de lepidoptere diurne (Lepidoptera, Rhopalocera) a Județului Hunedoara Romania. Considerații Ecologice Biolgice și Zoogeografice Buletin Infomativ al Societatii Lepideptorologice Române.

[B8257114] Capușe I., Kovács A . (1987). Catalogul colecției de lepidoptere László Diószeghy de la Muzeul Județean Covasna. Sfantu Gheorghe.

[B8257122] Caradja A. (1931). Beiträge zur Lepidopterofauna Grossrumäniens fűr Jahr 1930. Academia Română Membrii Secției de Științe București.

[B8257131] Chimișliu C. (1989). Colecția de lepidoptere "M. Peiu" conservată la Complexul Muzeal Județean Dolj Oltenia. Studii şi Comunicări Ştiinţele Naturii Muzeul Olteniei Craiova.

[B8257140] Chimișliu C. (2006). Lepidoptera (Insecta, Lepidoptera) from Romania preserved in the "Ion Firu" Entomological Collection from the Oltenia Museum Craiova. Entomologica Romanica Cluj-Napoca.

[B8257149] Chimișliu C. (2010). New data regarding the diversity of the Nymphalidae family (Insecta: Lepidoptera) in the Oltenia fauna. România (I). Biodiversitatea și Managementul Insectelor în Romania.

[B8257158] Chimișliu C. (2011). New data regarding the diversity of the Nymphalidae family (Insecta: Lepidoptera) in the Oltenia fauna, România (I). Volumul de lucrări al Simpozionului „Biodiversitatea şi Managementul Insectelor din România” Suceava.

[B8257167] Chimișliu C. (2012). Noi date privind diversitatea familiei Nymphalidae (Insecta: Lepidoptera) în fauna Olteniei, România. Buletinul Știinţific al Muzeului Naţional de Etnografie şi Istorie Naturală a Moldovei.

[B8257176] Choi J. B., Win N. Z., Han G. Y., Eun Y. C., Jinyong P., Jong K. P. (2021). Checklist of the family Nymphalidae (Lepidoptera: Papilionoidea) from Myanmar. Journal of Asia-Pacific Biodiversity.

[B8257187] Ciochia V., Barbu A . (1980). Catalogul colecției de Lepidoptere "Nicolae Delvig" a Muzeului Județean Brașov. Cumidava. Seria Științele Naturii.

[B8257214] Directive Council (1992). Habitats Directive 19. Convenţia de la Berna. Legea nr. 13 / 1993. https://legislatie.just.ro/Public/DetaliiDocumentAfis/3036.

[B8257196] Czekelius D (1897). Kritisches Verzeichnis der Schmetterlinge Siebenbűrgens. Siebenbürgischen verein für Naturwissenschaften zu Hermannstadt.

[B8257205] Czekelius D. (1898). Beiträge zur Schmetterlinsfauna Siebenbűrgens. Siebenbürgischen Verein für Naturwissenschaften zu Hermannstadt.

[B8257222] Fox R., Nigel A. D.B., E.B. Dennis, Heafeld R. T., Maclean I. M.D., Wilson R. J. (2019). Opinions of citizen scientists on open access to UK butterfly and moth occurrence data. Biodiversity and Conservation.

[B8257233] Karsholt O., Razowski J. (1996). The Lepidoptera of Europe a distributional checklist.

[B8257241] Lampert K . (1923). Die Grosschmetterlinge und Raupen Mitteleuropas Schreiber. Ed. 2. Esslingen und München.

[B8257250] Marcu A., Rákosy L. (2002). Catalogul coleției de lepidoptere Dr. Vladimir Olaru din Complexul Muzeal de Științele Naturii Galați.

[B8257258] Moise C. (2011). Lepidoptera (Insecta: Lepidoptera) in the collection of Eugen Worell from Natural History Museum of Sibiu, collected from "Dumbrava Sibiului" forest. Lucrări Știinţifice seria Horticultură "Ion Ionescu la Brad" Iaşi.

[B8257267] Moise C. (2011). Lepidoptera (Insecta: Lepidoptera) in the collection of Daniel Czekelius from Natural History Museum of Sibiu, collected from "Dumbrava Sibiului" Forest, Romania. Analele Universității din Oradea. Fascicula Biologie.

[B8257276] Moise C. (2011). Study on the Macrolepidoptera collected from the Dumbrava Sibiului forest existing within the collection of Dr. Viktor Weindel. Muzeul Olteniei Craiova. Studii şi Comunicări Ştiinţele Naturii.

[B8339958] Murariu Dumitru, Maican Sanda (2021). The Red Book of Invertebrates of Romania.

[B8257285] Niculescu E. V. (1965). Familia Nymphalidae (Insecta).

[B8257293] Popescu-Gorj A. (1960). Lépidoptères nouveaux ou rares pour la faune de la RPR. Travaux du Muséum National d’Histoire Naturelle “Grigore Antipa”.

[B8257302] Popescu-Gorj A. (1964). Catalogue de la colection de lépidoptères "Prof. A. Ostrogovich" du Museum d'Historie Naturelle "Grigore Antipa" București.

[B8257310] Popescu-Gorj A., Drăhia I. (1964). Noi cercetări privind fauna lepidopterelor din nordul Dobrogei. Academia RSR. Studii si Cercetări Biologice și Zoologice.

[B8336631] Rakosy Laszlo, Goia Marin (2021). The Lepidoptera of Romania: a Distributional Checklist.

[B8257319] Rákosy L., Goia M., Kovács Z. (2003). Verzeichnis der Schmetterlinge Rumäniens.

[B8257327] Rákosy L. (2013). Fluturii diurni din România, cunoaștere, protecție, conservare.

[B8336667] Rákosy László, Corduneanu Constantin, Crișan Andrei, Dincă Vlad, Kovács Sándor, Stănescu Mihai, Székely Levente (2021). Romanian Red List of Lepidoptera.

[B8257337] Schneider E. (1984). Die Gross-schmetterlinge der Sammlung “Dr. V. Weindel”–Ein beitrag zur faunistik der lepidopteren südsiebenbürgens und angrenzender gebiete. Studii şi Comunicări Ştiitele Naturii Muzeul Brukenthal Press Sibiu.

[B8257346] Schneider E. (1996). Zur Schmetterlingsforschung in Hermannstadt in den Jahren 1945-1985. Stapfia Press Viena.

[B8257355] Stancă-Moise C. (2017). Study on the Macrolepidoptera collected from Păltiniș (Sibiu County), existing within the collection of Dr. Viktor Weindel. Museum of Oltenia Craiova. Oltenia. Studies and Communications. Natural Sciences.

[B8257364] Stancă-Moise C. (2018). The species of macrolepidoptera collected from the Gușterița Hill, Sibiu, existing within the collection of dr. Viktor Weindel. Museum of Oltenia Craiova. Oltenia. Studies and Communications. Natural Sciences.

[B8257373] Stancă-Moise C. (2019). The species of macrolepidopctera collected from Cisnădioara and Cisnădie, county Sibiu, existing within the collection of dr. Viktor Weindel. Museum of Oltenia Craiova. Oltenia. Studies and Communications. Natural Sciences.

[B8257382] Stancă-Moise C. (2020). Forests and agricultural ecosystems pests (Lepidoptera), preserved in the entomological collections of the Natural History Museum In Sibiu (Romania). The Annals of Oradea University. Biology Fascicle.

[B8257391] Stancă-Moise C. (2021). *Zerynthiapolyxena* ([Dennis et Schiffermuller], 1775) and *Zerynthiacerisyiferdinandi* Stichel, 1907 (Lepidoptera, Papilionidae), in the collections of the Natural History Museum in Sibiu Romania. The Annals of Oradea University. Biology Fascicle.

[B8257400] Stancă-Moise C. (2022). Distribution of the genus *Vanessa* Fabricius, 1807 (Nymphalidae) in Sibiu (Romania) and its surroundings from 1904 to 1984. The Annals of Oradea University. Biology fascicle.

[B8257409] Stănescu M. (2005). The catalogue of the Ion Lăzărescu, collection of Lepidoptera (Insecta) from the "Grigore Antipa" National Museum of Natural History (Bucharest). Travaux du Muséum National d’Histoire Naturelle “Grigore Antipa”.

[B8257418] Ștefanescu C., Páramo F., Åkesson S., Alarcón M., Ávila A., Brereton T., Carnicer J., Cassar L. F., Fox R., Heliölä J., Hill J. K., Hirneisen N., Kjellén N., Kühn E., Kuussaari M., Leskinen M., Liechti F., Musche M., Regan E. C., Reynolds D. R., Ryrholm N., Schmaljohann H., Settele J., Thomas C. D., Van Swaay C., Chapman J. (2013). Multi-generational long-distance migration in insects: studying the painted lady butterfl y in the Western Palaearctic. Ecography.

[B8257449] Ștefanescu C., Ubach A., Wiklund C. (2021). Timing of mating, reproductive status and resource availability in relation to migration in the painted lady butterfly. Animal Behaviour.

[B8257458] Székely L. (1996). Lepidoptere (Fluturii) din sud-estul Transilvaniei (Romania).

[B8257466] Székely L. (2003). Istoricul cercetărilor lepidopterologice din sud-estul Transilvaniei. Lucrările Celei de a 6-a Conferinţe Naţionale Pentru Protecţia Mediului Prin Metode şi Mijloace Biologice şi Biotehnice şi a Celei de a 3-a Conferinţe Naţionale de Ecosanogeneză.

[B8257475] Székely L. (2004). Noutăți lepidopterologice din sud-estul Transilvaniei (Județul Brașov, Romania). Buletin Informativ Entomologic.

[B8257484] Szèkely L. (2008). The butterflies of Romania.

[B8257492] Török S., Cuzepan G. (2014). Butterfly (Insecta: Lepidoptera) hot spots in Sibiu county (Tansylvania, Romania). Brukenthal Acta Musei.

[B8257501] Van Swaay C. A.M., Warren M. (1999). Red data book of European butterflies (Rhopalocera). Nature and Environment. Strasbourg.

[B8257509] Wildman J. P., Ollerton J., Bourn N. A.D., Brereton T. M., Moore J. L., McCollin D. (2022). The value of museum and other uncollated data in reconstructing the decline of the chequered skipper butterfly *Carterocephaluspalaemon* (Pallas, 1771). Journal of Natural Science Collections.

[B8257520] Worell E. (1951). Contribuţii la cunoaşterea faunei coleopterelor şi lepidopterelor din Transilvania, mai ales din împrejurimile oraşului Sibiu. Academia Republicii Populare Romane. Buletin Știinţific Secţia Știinţe Biologice Agronomie Geologie Geografie Bucureşti.

